# Supplementary material for: The Long-Term Dietitian and Psychological Support of Obese Patients Who Have Reduced Their Weight Allows Them to Maintain the Effects
Source: Nutrients. 2021 Jun 11;13(6):2020. doi: 10.3390/nu13062020 (PMC8231289; doi:10.3390/nu13062020)
Supplement: Supplementary file 1 [file nutrients-13-02020-s001.zip › nutrients-1228823-supplementary.pdf]

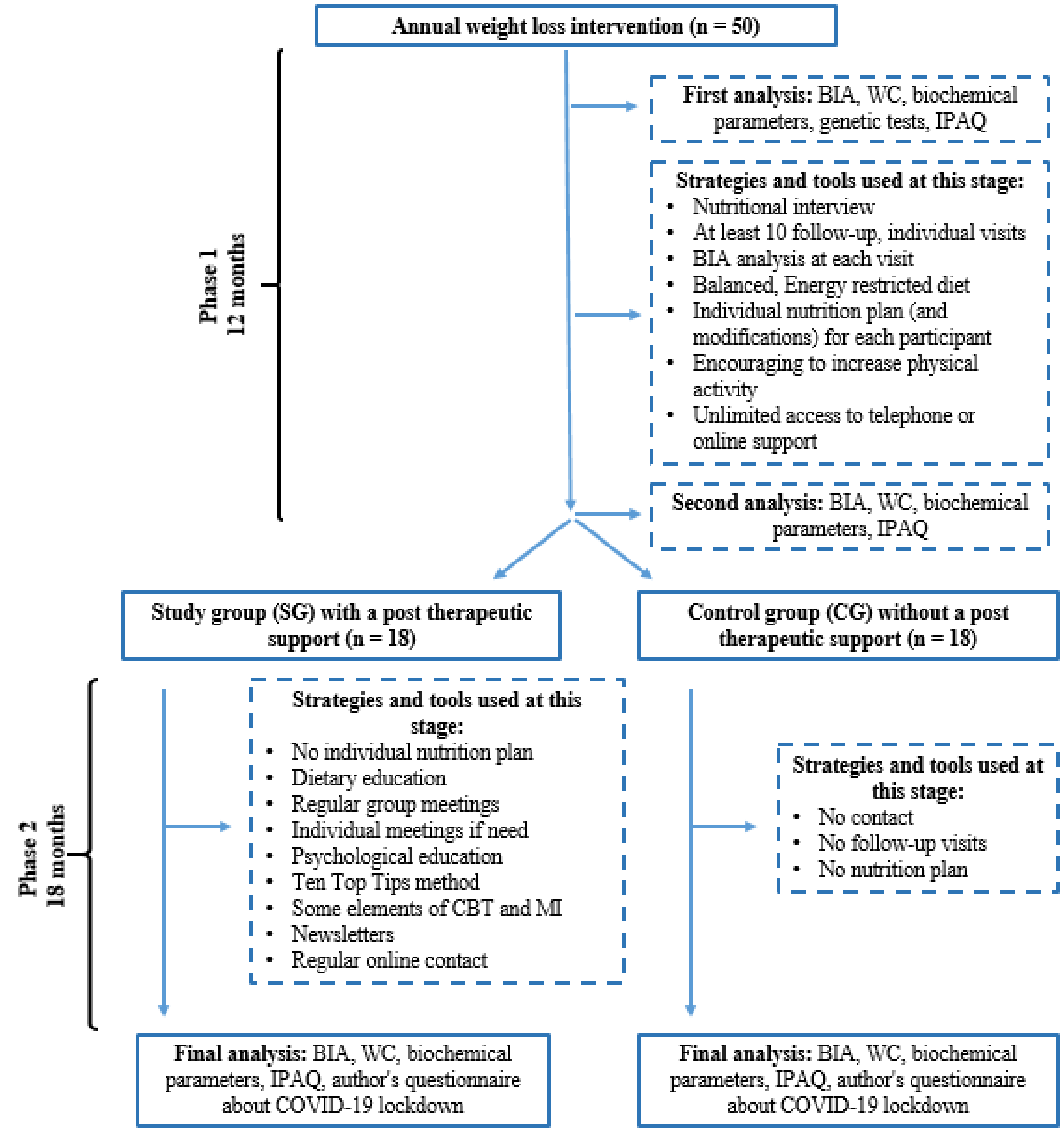

Figure S1. Scheme of the interventions used in Phase 1 and Phase 2.

Abbreviations: BIA (bioimpedance analysis), WC (Waist Circumference), IPAQ (International Physical Activity Questionnaire), CBT (cognitive behavioral therapy), MI (motivational interviewing)

Table S1. Patient's opinion about the study program and dietary patterns during the COVID-19 lockdown.

Question	SG (n = 18)	CG (n = 18)
**1) The support I received from a dietitian significantly has improved my nutritional knowledge**		
**a) Yes, definitely**	**56%**	**28%**
b) Yes, I think so	33%	44%
c) Unsure	11%	28%
d) No, not really	0%	0%
2) **I feel that the dietitian and psychologist took into account my circumstances and eating habits**		
**a)** Yes, definitely	89%	44%
**b)** Yes, I think so	11%	56%
c) Unsure	0%	0%
**d)** No, not really	0%	0%
3) **I will use in the future the obtained information**		
**a)** Yes, definitely	56%	72%
**b)** Yes, I think so	33%	28%
**c)** Unsure	11%	0%
**d)** No, not really	0%	0%
4) **If I were re-recruited, I would take part in this research again**		
**a)** Yes, definitely	89%	72%
**b)** Yes, I think so	11%	17%
**c)** Unsure	0%	11%
**d)** No, not really	0%	0%
5) **The nutrition care I received met my expectations**		
**a)** Yes, definitely	100%	83%
**b)** Yes, I think so	0%	17%
**c)** Unsure	0%	0%
**d)** No, not really	0%	0%
6) **The spoken and written information gave me lots of helpful hints**		
**a)** Yes	100%	83%
**b)** Unsure	0%	17%
**c)** No	0%	0%
7) **Pandemia COVID-19 negatively affects my weight loss process**		
**a)** Yes, definitely	44%	44%
**b)** Yes, I think so	39%	44%
**c)** No, I don't think so	11%	12%
**d)** No, definitely not	6%	0%
8) **Pandemia COVID-19 reduced my motivation for weight loss**		
**a)** Yes	66%	72%
**b)** Unsure	28%	28%
**c)** No	6%	0%
9. **During the last year, I have less controlled over my body weight**		
**a)** Yes	66%	100%
**b)** Unsure	28%	0%
**c)** No	`6%	0%
10. **I put off making more significant changes to the current diet until "after pandemic" time?**		
**a)** Yes	17%	11%
**b)** Unsure	56%	56%
**c)** No	27%	33%
11. **When you stopped seeing the dietitian, did you feel that the meetings had helped you with the changes you needed to make to your diet?**		

**a)** Yes	89%	28%
**b)** Unsure	11%	56%
**c)** No	0%	16%

12. **During the last year compared to the previous period (before the COVID-19 pandemic):**

	**SG (n=18)**			**CG (n=18)**		
	**Yes**	**Unsure**	**No**	**Yes**	**Unsure**	**No**
I eat more vegetables	22%	11%	67%	0%	17%	83%
My meals are more regular	28%	22%	50%	33%	11%	66%
I consume more homemade meals	89%	0%	11%	22%	50%	28%
I pay more attention to the caloricity of meals	56%	0%	44%	22%	11%	67%
I eat more snacks between main meals	33%	17%	50%	67%	11%	22%
I consume more unhealthy products (eg sweets, salty snacks, cakes, etc.)	61%	11%	28%	88%	6%	6%
I consume more products with a long expiration date	39%	39%	22%	17%	39%	44%
My present nutrition I consider as better	44%	12%	44%	17%	0%	83%
